# Therapeutic effects of Low intensity extracorporeal low energy shock wave therapy (LiESWT) on stress urinary incontinence

**DOI:** 10.1038/s41598-020-62471-4

**Published:** 2020-04-02

**Authors:** Cheng-Yu Long, Kun-Ling Lin, Yung-Chin Lee, Shu-Mien Chuang, Jian-He Lu, Bin-Nan Wu, Kuang-Shun Chueh, Chin-Ru Ker, Mei-Chen Shen, Yung-Shun Juan

**Affiliations:** 1 Department of Obstetrics and Gynecology, Kaohsiung Medical University Hospital, Kaohsiung, Taiwan; 2 Department of Obstetrics and Gynecology, Kaohsiung Municipal Hsiao-Kang Hospital, Kaohsiung, Taiwan; 3 Graduate Institute of Medicine, College of Medicine, Kaohsiung Medical University, Kaohsiung, Taiwan; 4 Graduate Institute of Clinical Medicine, College of Medicine, Kaohsiung Medical University, Kaohsiung, Taiwan; 5 Department of Urology, College of Medicine, Kaohsiung Medical University, Kaohsiung, Taiwan; 6 Department of Urology, Kaohsiung Municipal Hsiao-Kang Hospital, Kaohsiung, Taiwan; 7 Department of Urology, Kaohsiung Medical University hospital, Kaohsiung, Taiwan; 8 Translational Research Center, Cancer Center, Department of Medical Research, Kaohsiung Medical University, Kaohsiung, Taiwan; 9 Department of Pharmacology, Graduate Institute of Medicine, College of Medicine, Kaohsiung Medical University, Kaohsiung, Taiwan; 10 Department of Urology, Kaohsiung Municipal Ta-Tung Hospital, Kaohsiung, Taiwan

**Keywords:** Bioenergetics, Bladder

## Abstract

This study aimed to evaluate the therapeutic effects of Low intensity extracorporeal low energy shock wave therapy (LiESWT) on stress urinary incontinence (SUI). The investigation was a single-arm, open-label, multicentre study conducted in Taiwan. 50 female patients with SUI received LiESWT-treated with 0.25 mJ/mm^2^ intensity, 3000 pulses, and 3 pulses/second, once weekly for 4-weeks (W4) and 8-weeks (W8). The pad test, uroflowmetry, life quality questionnaires, and 3-day urinary diary measurement were performed before and after LiESWT intervention. The results revealed that 8-week of LiESWT treatment meaningfully improved urine leakage (pad test), maximum flow rate, post-voided residual urine, average urine volume, functional bladder capacity, urinary frequency, urgency symptom, and nocturia, which also persisted to show significant improvements at 1-month follow up (F1). Moreover, bothersome questionnaires scores were significantly improved at W4, W8, and F1 as compared to the baseline (W0). These results indicated that 8 weeks of LiESWT attenuated SUI symptoms on physical activity, reduced bladder leaks and overactive bladder (OAB), implying that LiESWT brought significant improvement in the quality of life. (ClinicalTrials.gov number, NCT04059133).

## Introduction

Stress urinary incontinence (SUI) is a prevalent urologic problem that is characterized by involuntary leakage of urine upon physical activity, such as exercise, exertion, sneezing, coughing, and lifting heavy objects, leading to affect a woman’s physical, psychological and social activity, and impact on her quality of life (QoL). SUI is a prevalent gyneco-urological problem worldwide, with an estimate as high as 40% in adult women with urethral sphincter deficiency^1,2^. Vaginal delivery, aging, obesity, and menopause are some known risk factors. SUI also causes great psychosocial and sexual distress that is both costly in terms of health care expense and the QoL. Chong and colleagues reported that approximately 13.12 billion US dollars were spent on SUI, including sanitary pads, disposable underwear, diapers, laundry, dry cleaning, treatment and diagnosis^3^.

A spectrum of management modalities is currently available: lifestyle intervention and pelvic floor muscle training might be effective for mild symptom; electro-stimulation, vaginal devices and urethral inserts are non-invasive and temporary symptom-control methods; bulking agents and botulinum injections are less invasive with short-term effectiveness; mid-urethral slings and colposuspension are corrective with long-term effectiveness^4^. Each of these methods has its strengths and limitations that should be chosen according to individual needs, characteristics, disease severity, and financial considerations. Meanwhile, Food and Drug Administration (FDA) has been skeptical regarding the long-term safety of synthetic mesh use in female urogynecology^5^. Following reclassification of the product to high risk group in 2016, all manufactures of surgical mesh intended for female pelvic organ prolapse were ordered to stop selling and distributing in April 2019. As a result of this, surgeons seek treatment alternatives for SUI, such as Low intensity extracorporeal low energy shock wave therapy (LiESWT) in the current study.

Clinical LiESWT (2000 to 3000 pulses in 0.20–0.25 mJ/mm^2^) was reported to enhance wound healing, promote angiogenesis^6^, reduce the level of oxidative stress, induce the releasing of vascular endothelial growth factor (VEGF), stimulate proliferation and differentiation of stem cells, and the effect of tissue regeneration^6,7^. A vast body of evidence has reported LiESWT being effective in treating tendon-bone junction diseases^8–10^, ischemic cardiovascular disorders^11–13^, skin wound healing^14,15^, chronic prostatitis/chronic pelvic pain syndrome (CP/CPPS)^16,17^, chronic injuries of soft tissues and erectile dysfunction^18–20^. With the applications of LiESWT prospers in various fields, speculation about its use in treating SUI emerged. Importantly, the advantages of LiESWT include therapies without medication or surgery, outpatient therapies, short treatment sessions, no anesthesia required, and non-invasive outpatient therapy.

The urethral sphincteric system is critical to maintain urinary continence mostly dependent on the urethral striated muscles, but the mucosa, smooth muscle, and vascular system also play an important role. While the molecular mechanism underlying the treatment effect of LiESWT on SUI is still unclear, Zhang *et al*. has shown that LiESWT with 0.10–0.13 mJ/mm^2^, 200 to 300 pulses improved bladder functions due to angiogenesis, reduced oxidative stress, and decreased inflammation reaction in rats with cyclophosphamide-induced acute interstitial cystitis^21^. Wu *et al*. demonstrated that in a vaginal balloon dilation induced SUI rat model, LiESWT treatment with energy flux density of 0.06 mJ/mm^2^, and 300 pulses at 3 Hz significantly increased urethral sphincter regeneration to restore urethral closure function, promoted VEGF expression and angiogenesis, and enhanced progenitor cell recruitment^22^. Furthermore, in the streptozotocin (STZ) -induced diabetic underactive bladder (UAB) rat model, the LiESWT was applied toward the pelvis with 0.02 mJ/mm^2^, and 400 shocks at 3 Hz for 4 weeks. The obtained data implied that LiESWT not only ameliorated UAB and urinary incontinence but also improved bladder function and urethral structure^23^. Thus, in the present study, we hypothesized that clinical application of LiESWT can attenuate bladder leaks, improve overactive bladder (OAB) and promote QoL.

## Results

### Functional analyses on physical indicators and serum parameters of studied subjects

**Figure 1:**
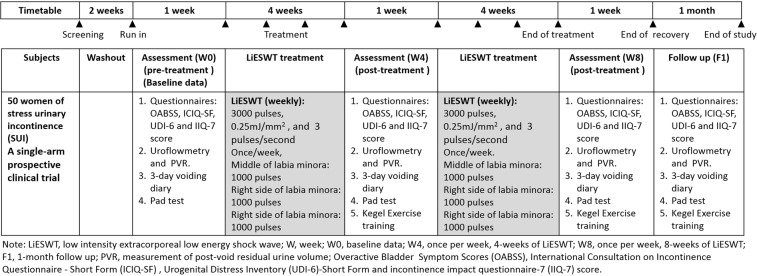
Timetable design for clinical trial of stress urinary incontinence (SUI).

Timetable design for the current clinical trial of SUI was shown in Fig. 1. A total number of 50 female subjects were enrolled in the NCT04059133 study group between 2018 and 2019. Participants aged 20–75 years who were diagnosed with involuntary leakage amount of urine ≧2 g on physical activity. The baseline characteristics of the SUI subjects at W0 were summarized in Table 1. The physical indicators, including age, height, weight, waistline, body mass index (BMI), systolic pressure, diastolic pressure, and mean arterial pressure (MAP), were shown in the normal range at W0 as listed in Table 1. The mean age of the enrolled 50 subjects was 54.00 ± 9.53 years old. Additionally, serum parameters including hemoglobin A1c (glycated hemoglobin; [HbA1C], blood sugar, liver function index (glutamate oxaloacetate transaminase [GOT] and glutamate pyruvate transaminase [GPT]), renal function index (Blood Urea Nitrogen [BUN] and creatinine), and lipid profile (triglycerides, cholesterol, low-density lipoprotein [LDL], and high-density lipoprotein [HDL]) were used to determine the baseline characteristics of SUI population. All serum parameters were also characterized for the normal range at W0 as listed in Table 1.

**Table 1:** Baseline characteristics of stress urinary incontinence (SUI) population.

Parameter	SUI (Mean ± SE)	Range
**Physical parameter**		
Female age (years)	54.00 ± 9.53	20–75
Height (cm)	158.22 ± 6.14	
Weight (Kg)	60.46 ± 8.17	
BMI (kg/m^2^)	24.15 ± 2.99	18.5–24
Waistline (cm)	84.83 ± 8.86	
Systolic pressure (mmHg)	122.55 ± 17.59	100–120
Diastolic pressure (mmHg)	74.51 ± 11.15	60–80
MAP	90.52 ± 12.54	70–110
**Serum parameter**		
HbA1C (%)	5.63 ± 0.42	4–6
AC sugar (mg/dl)	101.59 ± 1 2.0	65–109
BUN (mg/dl)	12.40 ± 3.12	8–20
Creatinine (mg/dl)	0.66 ± 0.09	0.44–1.03
GOT(AST) (IU/L)	24.42 ± 8.56	10–42
GPT(ALT) (IU/L)	24.96 ± 14.08	10–40
Triglycerides (mg/dl)	117.63 ± 35.42	35–160
Cholesterol (mg/dl)	200.55 ± 30.64	140–200
HDL (mg/dl)	58.21 ± 15.18	29–85
LDL (mg/dl)	119.43 ± 30.35	0–130

Note: BMI, body mass index; MAP, mean arterial pressure; GOT, glutamate oxaloacetate transaminase; GPT, glutamate pyruvate transaminase; LDL, low-density lipoprotein; HDL, high-density lipoprotein; Values are means ± SE. *p < 0.05; **p < 0.01 VS. baseline data (W0). N = 50.

### LiESWT decreased bladder leaks by pad test

**Figure 2:**
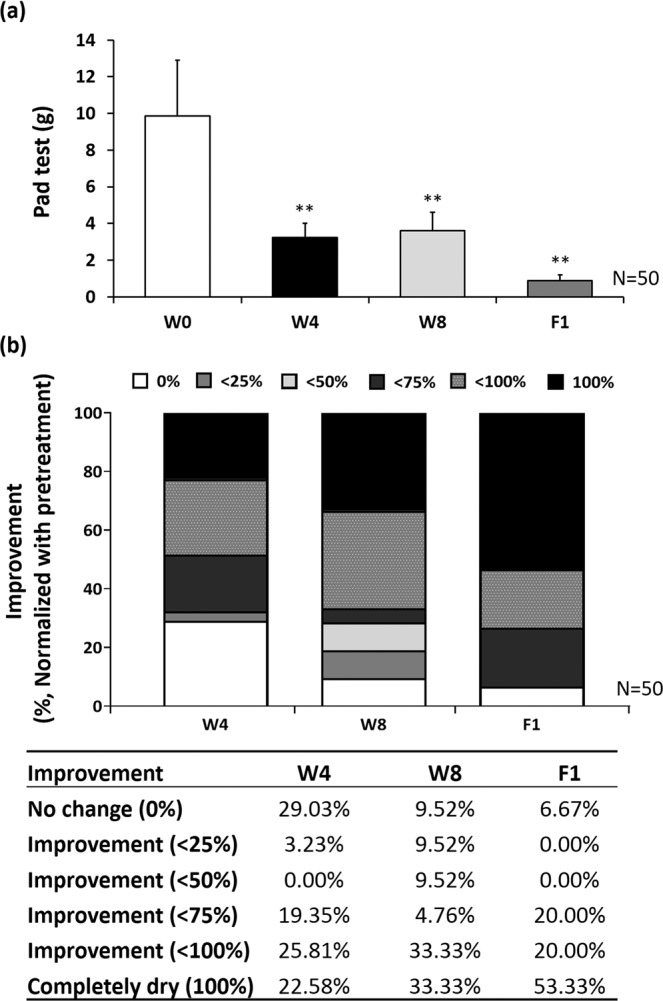
LiESWT decreased bladder leaks by pad test. (**a**) Pad test of study population for stress urinary incontinence at 4-week (W4), 8-week (W8), and 1-month follow up (F1). Values are means ± SE. N = 50. **p < 0.01 compared to the baseline (W0) by paired t-test. (**b**) The percentage of improvement at W4, W8, F1 after LiESWT treatment normalized with pre-treatment W0. N = 50.

The involuntary bladder leakage of urine on physical activity was examined by pad test performance. Pad test data revealed that the amount of bladder urine leakage by pad test was meaningfully reduced from 9.85 ± 3.06 to 3.23 ± 0.78 (*p* < 0.01), 3.60 ± 1.01 (*p* < 0.01) and 0.89 ± 0.31 (*p* < 0.01) grams at 4-week (W4), 8-week (W8), and 1-month follow up (F1), respectively **(**Table 2 and Fig. 2a**)**. At the end of 4-week and 8-week treatment, a significant improvement in urine leakage was observed. Moreover, 67.74% of women was reported to have moderate to better improvement (>50%) after 4-week therapy, and the proportion was increased to 71.42% after 8 weeks of LiESWT treatment, and 93.33% at F1 post-treatment **(**Fig. 2b**)**.

**Table 2:** Urodynamic parameters of study population for stress urinary incontinence (SUI).

Parameter	SUI (Mean ± SE)			
	W 0	W 4	W8	F1
**Pad test (g)**	9.85 ± 3.06	3.23 ± 0.78**	3.60 ± 1.01**	0.89 ± 0.31**
**Uroflowmetry data**				
Voided urine volume (ml)	354.59 ± 24.59	352.55 ± 25.57	360.25 ± 29.59	367.98 ± 44.26
Maximum flow rate (Qmax) (ml/sec)	33.89 ± 4.67	32.91 ± 2.88	34.48 ± 4.26	35.96 ± 3.74
Post voided residual (PVR) (ml)	48.54 ± 8.59	35.66 ± 5.87*	28.81 ± 6.57**	26.00 ± 6.62**
**3-day urinary diary data**				
Intake (ml)	1784.25 ± 88.53	1852.80 ± 96.72	1738.79 ± 85.37	1729.48 ± 118.51
Output (ml)	1751.62 ± 91.68	1819.70 ± 89.98	1771.05 ± 84.43	1731.77 ± 114.13
Average urine volume (ml)	219.70 ± 11.37	229.31 ± 10.49	237.01 ± 11.56*	232.59 ± 15.21
Functional bladder capacity (ml)	363.93 ± 15.54	383.47 ± 19.00*	386.13 ± 24.62*	379.16 ± 30.76*
Urinary frequency (times/24hrs)	8.54 ± 0.31	7.38 ± 0.38	6.15 ± 0.40*	7.39 ± 0.46
Urgency (times)	1.95 ± 0.31	0.97 ± 0.24**	0.83 ± 0.25**	0.78 ± 0.26**
Nocturia (times)	1.12 ± 0.12	0.84 ± 0.13	0.76 ± 0.14*	0.74 ± 0.22*

Note: W, week; W0, baseline data; W4, once per week, 4 weeks of LiESWT; W8, once per week, 8 weeks of LiESWT; F1, 1-month follow up; Valus are means ± SE. *p < 0.05; **p < 0.01 VS. baseline data (W0). N = 50.

### LiESWT improved urodynamic parameters

The analysis of urodynamic parameters was performed using uroflowmetry (voided urine volume and maximum uroflow rate [Qmax]), post-voided residual urine (PVR) and 3-day urinary diary at the W0, W4, W8 of LiESWT treatment, and F1 follow up after LiESWT. The results were shown in Table 2. There was no significant difference in the mean of voided urinary volume and Qmax. However, the mean of PVR was noticeably decreased from 48.54 ± 8.59 ml (W0) to 35.66 ± 5.87 ml (*p* < 0.05), 28.81 ± 6.57 ml (*p* < 0.01), and 26.00 ± 6.62 ml (*p* < 0.05) at W4, W8 and F1, respectively. These findings indicated that SUI subjects exhibited a significant decrease in PVR after LiESWT treatment.

The analysis of 3-day urinary diary data was characterized in Table 2, which revealed no meaningful difference in the amount of water intake and urine output among different groups. However, 8-week of LiESWT treatment showed significant increases in the average urine volume from 219.70 ± 11.37 ml to 237.01 ± 11.56 ml (*p* < 0.05). According to the 3-day urinary diary data, the functional bladder capacity was also significantly promoted from 363.93 ± 15.54 ml to 383.47 ± 19.00 ml (*p* < 0.05), 386.13 ± 24.62 ml (*p* < 0.05) and 379.16 ± 30.76 ml (*p* < 0.05) at W4, W8 and F1, respectively. The mean times of the urinary frequency (times/24hrs) was reduced from 8.54 ± 0.31 to 6.15 ± 0.52 (*p* < 0.05) times at W8. The mean times of the urgency was suppressed from 1.95 ± 0.31 times to 0.97 ± 0.24 times (*p* < 0.01), 0.83 ± 0.25 times (*p* < 0.01) and 0.78 ± 0.26 times (*p* < 0.01) at W4, W8 and F1, respectively. Moreover, the nocturia was noticeably decreased from 1.12 ± 0.12 times to 0.76 ± 0.14 times (*p* < 0.05) and 0.74 ± 0.22 times (*p* < 0.05) at W8 and F1, respectively. Based on the 3-day urinary diary, the mean value of daytime urinary frequency, urgency and nocturia was also reduced at 8-week of LiESWT.

### LiESWT improved SUI symptoms and promoted the QoL

**Figure 3:**
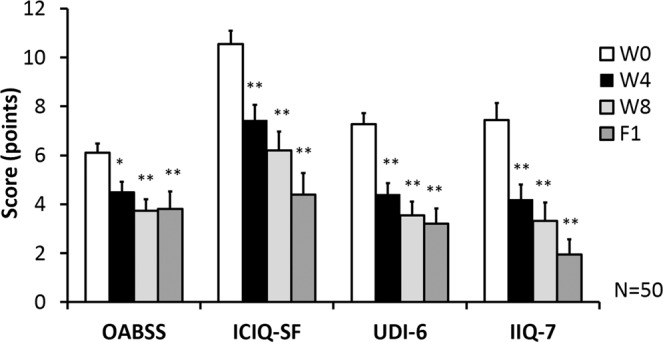
The change of stress urinary incontinence (SUI) symptoms and bothersome questionnaire scores after LiESWT treatment. The bothersome questionnaires scores included overactive bladder Symptom Scores (OABSS), International Consultation on Incontinence Questionnaire-Short Form (ICIQ-SF), Urogenital Distress Inventory (UDI-6)-Short Form, and Incontinence Impact Questionnaire-7 (IIQ-7) score at 4-week (W4), 8-week (W8), and 1-month follow up (F1) after LiESWT treatment. Values are means ± SE. N = 50. *p < 0.05; **p < 0.01 compared to the baseline (W0) by paired t-test.

We also investigated the relationship between LiESWT treatment and overactive bladder (OAB) as well as involuntary leakage of urine on physical activity. Subjective evaluation using Overactive Bladder Symptom Scores [OABSS], International Consultation on Incontinence Questionnaire-Short Form [ICIQ-SF], Urogenital Distress Inventory [UDI-6]-Short Form, and Incontinence Impact Questionnaire-7 [IIQ-7] score questionnaires revealed significant improvement at the end of 4-week, 8-week treatment and 1-month follow up after the last treatment (Fig. 3). The OABSS score was also significantly lessened from 6.10 ± 0.38 to 4.49 ± 0.43 (*p* < 0.05), 3.74 ± 0.46 (*p* < 0.01) and 3.80 ± 0.72 (*p* < 0.01), at W4, W8 and F1, respectively. The ICIQ-SF score was noticeably decreased from 10.54 ± 0.56 to 7.44 ± 0.62 (*p* < 0.01), 6.20 ± 0.77 (*p* < 0.01) and 4.40 ± 0.88 (*p* < 0.01) at W4, W8 and F1, respectively. The UDI-6 score was lessened from W0 7.28 ± 0.44 to 4.40 ± 0.46 (*p* < 0.01), 3.54 ± 0.57 (*p* < 0.01) and 3.20 ± 0.63 (*p* < 0.01) at W4, W8 and F1, respectively. The IIQ-7 score was decreased from W0 7.44 ± 0.69 to 4.20 ± 0.60 (*p* < 0.05), 3.31 ± 0.77 (*p* < 0.01) and 1.95 ± 0.61 (*p* < 0.05) at W4, W8 and F1, respectively. In examining questionnaire data, it was found that the OAB symptoms and bothersome questionnaire scores (OABSS, ICIQ-SF, UDI-6, and IIQ-7) were significantly improved at W4, W8, and F1, as compared to W0 (*p* < 0.01) **(**Fig. 3**)**. According to questionnaire scores and the 3-day urinary diary data, the results indicated that LiESWT treatment improved bladder urine leakage and bladder activity symptoms, including urinary frequency, nocturia, urgency, and urgency incontinence.

### Safety of LiESWT treatment

For the safety concern, LiESWT treatments were well tolerated by all the subjects in this study. No significant adverse effect associated with LiESWT, such as intolerable pain, hematuria or skin ecchymosis, was reported.

### Short graphic abstract of a proposed model of the potential effect of LiESWT

**Figure 4:**
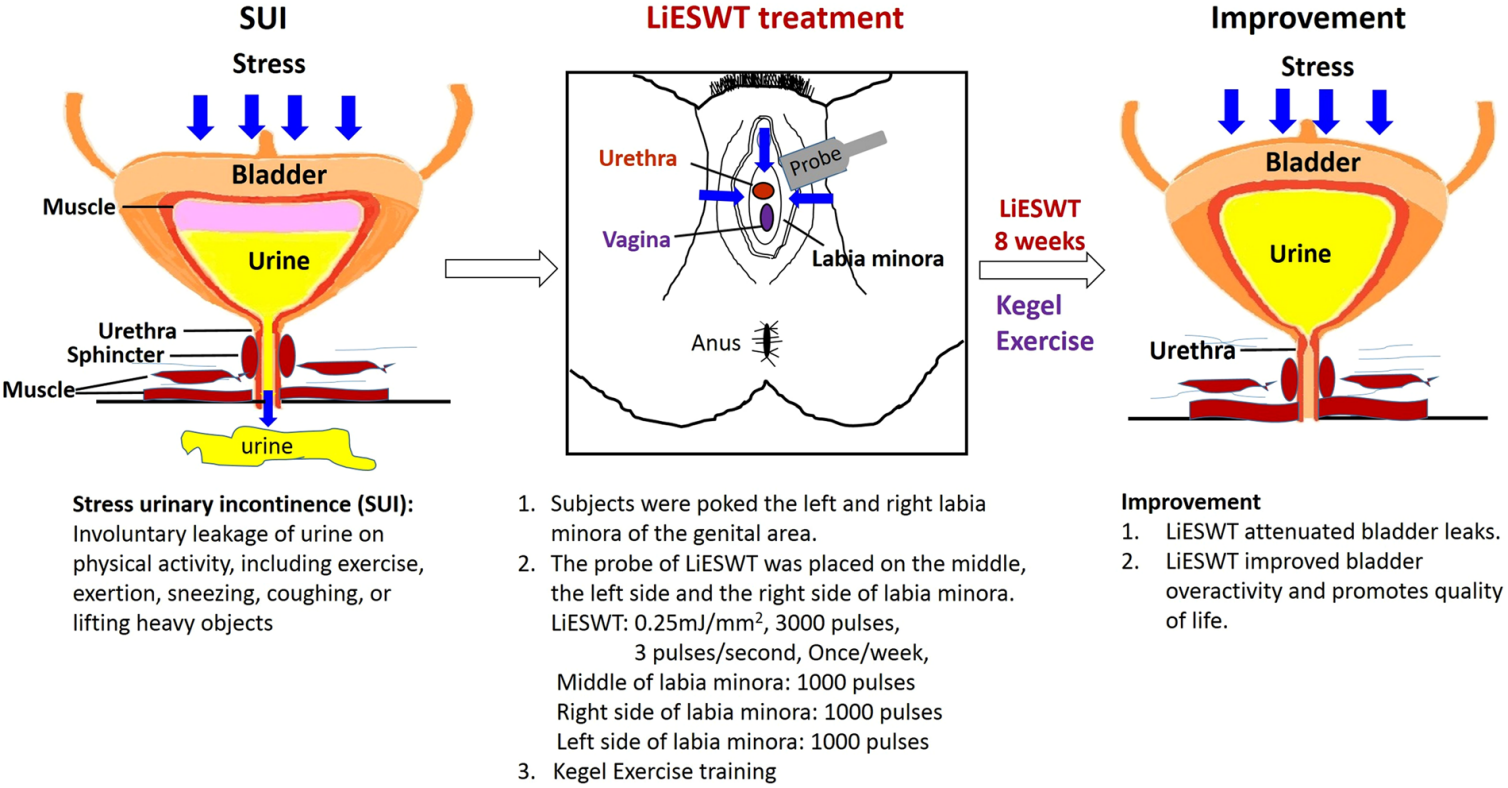
Short graphic abstract of study for a proposed potential effect of LiESWT. SUI, stress urinary incontinence; LiESWT, low intensity extracorporeal low energy shock wave therapy.

The above findings led to a proposed model of the potential effect of LiESWT on bladder leakage of SUI subjects, as presented in Fig. 4. The urethral sphincteric system is critical to maintain urinary continence, mostly depending on the urethral striated muscles, but the mucosa, smooth muscle, and vascular system also play an important role, where SUI is defined as involuntary leakage of urine on physical activity, including exercise, exertion, sneezing, coughing, or lifting heavy objects. The clinical LiESWT was applied with 0.25 mJ/mm^2^ intensity, 3000 pulses of shocks, and frequency of 3 pulses/second with the probe of LiESWT placed on the middle, the left side and the right side of the labium minor. The results revealed that LiESWT attenuated the syndrome of SUI, including bladder leaks, urinary incontinence, urgency, frequency, and nocturia after 8 week treatment, improved overactive bladder (OAB), which brought meaningful improvement in QoL.

## Discussion

Our investigation demonstrated that at the end of 4-week treatment, there were significant improvements in PVR, functional bladder capacity, urine leakage, and urgency symptom. Besides, at the end of 8-week treatment, significant improvements were observed in PVR, functional bladder capacity, urine leakage, urinary frequency, urgency symptom, and nocturia. Moreover, PVR, functional bladder capacity, urine leakage, urgency symptom, and nocturia persisted to show significant improvements at 1-month follow up. The above findings indicated that clinical application of LiESWT attenuated SUI symptom on physical activity, reduced bladder leaks and overactive bladder (OAB), improved pelvic floor tissue regeneration and promoted QoL.

Clinical trials on the safety and efficacy profile of LiESWT in treating female SUI are still not completely clear. Clinically, LiESWT has emerged in recent years to treat (1) chronic pelvic pain syndrome (CPPS) (0.10–0.25 mJ/mm^2^, 3000 pulses, once/week, 4 weeks) to improve pain, bladder voiding and QoL^17,24–27^; (2) erectile dysfunction (ED) (0.10–0.25 mJ/mm^2^, 3000–6000 pulses, once/week, 4–8 weeks) to increase penile hemodynamics and induce penile tissue regeneration^18,28,29^. In this study, the recruited subjects had normal BMI, blood pressure, renal function, liver function, blood sugar level, and lipid profiles, thus eliminating the potential confounding factors **(**Table 1**)**. LiESWT treatment (0.25 mJ/mm^2^, 3000 pulses, once/week) for 4 to 8 weeks demonstrated significant improvement in incontinence symptoms. Sixty-eight percent of the study cohort reports showed moderate or better symptom improvement at the end of 4-week treatment. This number was increased to 72% after 8-week treatment and 93% at 1-month follow up post-treatment **(**Fig. 2**)** with LiESWT, as demonstrated by significant improvement of symptom scores using OABSS, ICIQ-SF, UDI-6, and IIQ-7 **(**Fig. 3**)**. This investigation was the first clinical study to report LiESWT’s treatment on female SUI. However, the study is limited by short-term follow up.

We investigated the relationship between LiESWT treatment and SUI as well as involuntary leakage of urine on physical activity. Subjective evaluation using OABSS, ICIQ-SF, UDI-6, and IIQ-7 questionnaires appraised the incontinence improvement and changes of life quality of urinary bothersome before and after LiESWT treatment, owing to clinical subjects who were diagnosed SUI alone or SUI combined with OAB. In order to clarify the effects of LiESWT, detailed records and statistics must be made. The detailed analysis presented potential treatment effects of LiESWT for SUI alone or mixed UI patients.

Since it was not easy to obtain bladder tissues in clinical trials, animal experiments were carried out to understand the molecular mechanism of SUI and LiESWT therapeutic effect. In rat animal model, using oligo microarray analysis showed that the expression of genes involved in the inflammation (Smad2, involved in the signaling pathway of the transforming growth factor β [TGF-β]), smooth muscle regulation (regulator of G-protein signaling 2 [RGS2]) and collagen metabolism (matrix metalloproteinase 13 [MMP13]) was significantly increased in the parturition-induced SUI rats^30^. Zhang *et al*. showed that LiESWT with 0.10–0.13 mJ/mm^2^ and 200 to 300 pulses improved bladder functions, due to angiogenesis, reduced oxidative stress and decreased inflammation reaction in rats with cyclophosphamide-induced acute interstitial cystitis^21^. Moreover, the effects of LiESWT with 0.12 mJ/mm^2^ and 300 pulses suppressed overactive bladder and bladder pain by activating the expression of IL-6, NGF, and COX-2 in rats with cyclophosphamide-induced interstitial cystitis^31^. Wu *et al*. indicated that the beneficial effects of LiESWT with an energy flux density of 0.06 mJ/mm^2^ and 300 pulses at 3 Hz significantly increased urethral muscle regeneration to restore urethral closure function, promoted VEGF expression and angiogenesis, and enhanced progenitor cell recruitment in a vaginal balloon dilation (VBD) induced SUI rat model^22^. Furthermore, in the streptozotocin (STZ) -induced diabetic underactive bladder (UAB) rat model, the LiESWT was applied toward the pelvis with energy flux density of 0.02 mJ/mm^2^, and 400 shocks at 3 Hz for 4 weeks. It was found that LiESWT ameliorated UAB and urinary incontinence, and improved bladder function and urethral structure^23^. Additionally, understanding the biological effects of urethral striated muscle cells may expand understanding of urethral anatomy and increase treatment options for SUI. PERK/ATF4 pathway was involved in myotube formation, and rat myoblast cells were activated by LiESWT to form myotubes. The finding suggested that the effect of utilizing LiESWT stimulates urethral myogenesis through PERK/ATF4 pathway^32^. This information may help to further refine the use of LiESWT in the clinical trial of medicine.

SUI is a common and disturbing disease with a spectrum of management modalities, including pelvic floor exercise, biofeedback training, electrostimulation, vaginal laser therapy, and bulking agent injections. The various options imply that no single effective treatment could be universally recommended to the patients. Using surgical intervention that augments urethra stability with native tissue or synthetic mesh seems to work well. Consensus statement of the European Urology Association acknowledged that synthetic slings for SUI are effective with acceptable morbidity in 2017^33^. However, slings implantation still poses surgical risks and unwanted consequences, although few in number. Souders *et al*. analyzed more than 70,000 legal claims against synthetic mesh or sling use from 2000 to 2014, and found that the majority (63%) was related to SUI alone, from the retropubic sling procedures^34^. Reported complications included bladder perforation, hemorrhage, bowel injury, vaginal extrusion, de novo urgency, urinary tract infections, and voiding dysfunction, with incidence of 4.3% to 75.1%^35^. These concerns finally led to FDA ban on selling and distributing such products in the United States in 2019, thus warranting further search for treatment options that are both effective and safe.

For patient-centered care in our hospital, SUI patients were educated to do Kegel exercise as a lifestyle modification. If there is no obvious improvement after 3 months of conservative treatment, then LiESWT treatment was advised for those SUI subjects. Therefore, our SUI subjects received both LiESWT and Kegel exercise. Kegel exercise can strengthen pelvic floor muscle and enhance elasticity, which supports the uterus, bladder, small intestine and rectum to prevent leaking urine. Several previous studies showed Kegel exercise takes more than sixth month to make significant improvement. Kegel Exercise is also less effective for moderate to severe incontinence patients^36,37^. In the present study, improvement of SUI could be observed as early as 4 week of LiESWT in either moderate to severe SUI groups. Therefore, we proposed that LiESWT may play the major role in SUI improvement, while Kegel exercise plays as adjuvant for LiESWT.

The pad test is a simple and non-invasive procedure for quantifying the degree of urine leakage and evaluating the effect of LiESWT treatment. Performing the pad test with a starting volume and functional bladder capacity might enhance the accuracy, but the data supporting this assumption is still inconclusive. The volume of urine leakage does not always relate to the degree of bothering. Therefore, pad tests would be interpreted in conjunction with clinical symptoms, self-assessment questionnaires, and physical exams.

Although Kegel exercise was recommended by the International Continence Society, there are no standard exercise parameters of muscle contraction and relaxations, such as frequency, duration, repetitions, and the positions. Without immediate clinical improvement and cannot check their own exercise posture leading patients to give up Kegel exercises easily^38^. The right approach is more important than using an assisting device. Therefore, a standardized guideline for Kegel exercises needs to be developed since Kegel exercise must be done consistently throughout life, which may help to manage menopausal urinary incontinence. The type of pad test selected is based on goals.

The current study has several limitations. First, because this study was a single-arm study which lack of validation with randomized controlled and the small number of patients. Population benefiting from LiESWT treatment remains to be confirmed in larger prospective studies. Second, this investigation has only short-term follow up outcomes. Therefore, the durability of these effects is still unclear. Lastly, the bladder tissues were not easy to obtain in clinical trials. Thus, the biomarker analysis (eg, inflammation, angiogenesis, tissue repair and regeneration-related gene) was not comprehensive in our study. The potential molecular mechanism of SUI and LiESWT therapeutic effect needed to be further study by using animal experiments.

In summary, the current study has demonstrated treatment efficacy using LiESWT for female SUI in a short-time follow up. It is a promising alternative to surgical interventions with rejuvenating and curative properties to the weakened pelvic floor or external urethral sphincter. Further studies are called for its long-term efficacy and potential applications in SUI. The mechanism of LiESWT or its time course on human and rat treatment needs to be elucidated in future studies, and the LiESWT protocol likely requires future adjustment. Moreover, the therapeutic effects of LiESWT on anti-inflammation, angiogenesis, tissue repair and regeneration to the urethra, perineum and bladder should also be further investigated.

## Materials and Methods

### Eligibility of subject

This clinical trial was a single-arm, open-label, prospective study and performed with the approval of the institutional review board of a tertiary medical center. All participants provided informed consent, as approved by the Kaohsiung Medical University Hospital Institutional Review Board and was adhered to the Declaration of Helsinki (clinical trial registration No. KMUHIRB-F(II)-20180010), before entering the study and had a complete medical history and physical examination in the office of hospital. This study was also registered at clinicaltrials.gov (NCT04059133) and the date of registration was August 16, 2019. Female subjects aged 20–75 years who were diagnosed with SUI for more than 3 months. Major exclusion criteria included the followings: (1) urinary tract infection detected at screening, recurrent urinary tract infections (more than 3 episodes in the past 3 months), (2) comorbidities relevant to OAB (diabetes mellitus, spinal cord injury, stroke or neurogenic diseases), (3) severe cardiovascular diseases, (4) coagulopathy, (5) liver failure, (6) renal failure, (7) chronic urinary inflammation (interstitial cystitis, urethral syndrome or painful bladder syndrome), drug or alcohol abuse in the past 12 months, (8) lower urinary tract surgeries in the past 6 months, (9) perineal operations, intravesical injection, irradiation, shockwave or electrostimulation in the past 12 months, (10) urinary catheterization, urologic malignancy, gross hematuria, significant bladder outlet obstruction, kidney stones, chronic pelvic pain, or inability to comprehend or comply with instructions.

### Physical indicators and biochemical parameters of study subjects

The physical and serum parameters of metabolic syndrome were associated with the symptoms of SUI^39^. We analyzed the physical indicators, including age, height, weight, waistline, body mass index (BMI), systolic pressure, diastolic pressure, and mean arterial pressure (MAP). Additionally, we also examined biochemical parameters including hemoglobin A1c (glycated hemoglobin; HbA1C), blood sugar, glutamate oxaloacetate transaminase (GOT) and glutamate pyruvate transaminase (GPT) for liver function index, Blood Urea Nitrogen (BUN) and creatinine for renal function index, lipid profile on triglycerides, cholesterol, low-density lipoprotein (LDL), and high-density lipoprotein (HDL) to investigate the baseline characteristics of SUI population.

### Pad test for the evaluation of SUI

The pad test was applied as a non-invasive diagnostic to quantify the severity of SUI^40^. The purpose of pad test in this study was to evaluate the effect of LiESWT on reducing urinary incontinence symptoms in women with SUI. Previously reported studies recommended a volume equivalent to approximately 60–80% of the functional bladder capacity^40^. According to 3-day urinary diary data before the pad test and LiESWT, the functional bladder capacity of all subjects was determined. For the pad test, women subjects were asked to drink 1000 ml of water. A weighted pad was wearing after filling the bladder to 60–80% of the functional bladder capacity by using bladder scan sonography. In that way, the pad test could be standardization with lessen deviation. The subjects should perform physical activities (stair climbing, jumping, coughing), then the pad was weighed again. After wearing pad, the subjects were instructed to exercise for 30 minutes (stair climbing (30×), coughing vigorously (10×), jumping, running (1 minute), and washing hands in running water (1 minute)). The weights before and after exercise were used to calculate the pad absorption. For the pad test, an increase of <2 g was represented as slight incontinence, mild incontinence (2–10 g), moderate incontinence (11 to 50 g), and severe incontinence (>50 g)^41^. The percentage (%) of improvement was calculated at 4-week (W4), 8-week (W8), and 1-month follow up (F1) after LiESWT treatment and the results were normalized with pre-treatment baseline data (W0).

### Procedure and medical information of LiESWT

Subjects were informed of their treatment modalities including the required consent to join this study and once weekly LiESWT for 8 weeks and follow up at 4-week after completing the course of treatment **(**Fig. 1**)**. Our instrumentation was the DUOLITH SD1-TOP focused shock wave system (STORZ MEDICAL EvoTron^TM^, GA). The LiESWT was applied with 0.25 mJ/mm^2^ intensity, 3000 pulses of shocks, and frequency of 3 pulses/second, as modified from previous reports^9^. Subjects were poked at the left and right labia minora of the genital area, and the applicator was gently placed on the middle, the left side and the right side of the labia with 0.25 mJ/mm^2^ intensity and 1000 pulses of shocks individually.

### Procedure of Kegel exercise

The purpose of Kegel exercise strengthens the pelvic floor muscles and improving elasticity, which supports the uterus, bladder, small intestine and rectum to reduce urinary incontinence symptoms and prevent leaking urine in women with SUI. We educated patients how to identify pelvic floor muscles and tighten them, and to make sure pelvic muscle contractions. Initially, subjects tried to contract pelvic muscle for three seconds at a time and relaxed for a count of three. They gradually increased the length of contractions and relaxations, worked up to 10-second contractions as well as relaxations. Kegel exercise plays the role of adjuvant therapy in combination with LiESWT.

### Uroflowmetry and measurement of PVR volume

Before the treatment of LiESWT, uroflowmetry and PVR amount were checked to rule out voiding dysfunction. Uroflowmetry was served as a noninvasive screening test for selecting patients who should undergo more sophisticated urodynamic studies. In this study, voided urine volume and Qmax were recorded. Measurement of PVR, the amount of residual urine in the bladder after a voluntary void, was performed by Verathon BVI 9400 Bladder Scanner (Radiance Medical Systems, Kuala Lumpur, MALAYSIA). These examinations helped evaluate the therapeutic effect of LiESWT at W0, W4, and W8 of LiESWT, and F1 after LiESWT.

### Questionnaires for subjective evaluation

Subjective evaluation using OABSS, IICIQ-SF, UDI-6, and IIQ-7 score questionnaires to evaluate the incontinence improvement and life quality of urinary bothersome after treatment. Among these questionnaires, OABSS questionnaire was applied for evaluating OAB symptoms, including daytime frequency, nocturia, urgency, and urge incontinence^42^. ICIQ-SF questionnaire was used for evaluation the severity of urinary loss and quality of life for subjects with urinary incontinence^43^. UDI-6 and IIQ-7 questionnaires were short forms to assess quality of life and symptoms severity for urinary incontinence in women^44^.

### Therapeutic efficacy assessment for LiESWT

To analyze the effects of LiESWT, the primary endpoints were change in pad test and questionnaires, and the secondary endpoints were change in uroflowmetry, PVR, and 3-day urinary diary at W0, W4 and W8 of LiESWT and F1 after LiESWT. SUI questionnaires included overactive bladder questionnaire short form (OABSS, ICIQ-SF, UDI-6, and IIQ-7 scores). Moreover, uroflowmetry and PVR measurements were performed to assess the urodynamic parameters at W0, W4, W8, and F1. The primary endpoint was the mean change in SUI symptoms and urodynamic parameters from the baseline to the end-of-treatment.

### Statistical analysis

Questionnaires (OABSS, ICIQ-SF, UDI-6, and IIQ-7 scores), pad test, uroflowmatry (voided urine volume and Qmax), PVR, and 3-day urinary diary were used to assess the efficacy and safety of the pre- and post- treatments performed with LiESWT on SUI subjects. Quantitative data were represented as mean ± standard error of mean (SEM). In order to clarify the effect of LiESWT therapy on SUI, we compared the pre- and post-treatment scores (W4 vs. W0, W8 vs W0, F1 vs W0) for intragroup of patients, not between groups of patients. Therefore, Paired t-test was used to perform a repeated measurement analysis for intragroup before/after treatment and to calculate p-values for comparison^45,46^ in single-arm clinical trial of the current study. For all statistical analyses, *p* < 0.05 was considered statistically significant. All statistical analyses were performed using SAS 9.3 (SAS Institute, Cary, NC, USA).
